# SARS-CoV-2-derived fusion inhibitor lipopeptides exhibit highly potent and broad-spectrum activity against divergent human coronaviruses

**DOI:** 10.1038/s41392-021-00698-x

**Published:** 2021-08-03

**Authors:** Yuanmei Zhu, Danwei Yu, Yue Hu, Tong Wu, Huihui Chong, Yuxian He

**Affiliations:** NHC Key Laboratory of Systems Biology of Pathogens, Institute of Pathogen Biology, Chinese Academy of Medical Sciences and Peking Union Medical College, Beijing, China

**Keywords:** Industrial microbiology, Drug discovery

**Dear Editor**,

Most members of coronaviruses (CoVs), including four annually circulating human CoVs (HCov-229E, HCoV-OC43, HCoV-NL63, and CoV-HKU1), infect the respiratory tract of mammals and cause mild illness; however, zoonotic CoVs can cross the species barrier from animal reservoirs and lead to epidemics with high morbidity and mortality in humans. Highly pathogenic severe acute respiratory syndrome CoV (SARS-CoV) and Middle East respiratory syndrome CoV (MERS-CoV) emerged in 2002 and 2012, respectively. The current global COVID-19 epidemic was caused by SARS-CoV-2, which is a novel CoV genetically close to SARS-CoV.^1^ It is extremely urgent to develop therapeutics and prophylactics for combating SARS-CoV-2, and ideally, a drug or vaccine will possess broad-spectrum efficacy against divergent CoVs.

Like other CoVs, SARS-CoV-2 infection requires membrane fusion between the viral envelope and cell membrane, which is mediated by viral spike (S) glycoprotein composed of receptor-binding S1 and fusogenic S2 subunits (Fig. 1a). Typically, a six-helical bundle structure is formed by two heptad repeat domains (HR1 and HR2) in S2, juxtaposing the viral and cellular membranes for fusion. Previous studies demonstrated that peptides derived from the HR2 sequence can competitively bind to the viral HR1 domain thus exerting antiviral activity, and that lipid conjugation to HR2 peptides is a viable strategy to improve the antiviral potency.^2,3^ While a native HR2 peptide can block direct entrance from the cell surface but not entrance via the endosomal pathway, lipopeptides also enable activity against viruses that do not fuse until they have been taken up via endocytosis. We previously developed a group of HIV-1 fusion inhibitory lipopeptides with ultrapotent activity, with one being advanced to clinical trials (NCT04592315). In an immediate response to the COVID-19 outbreak, we applied our expertise to develop fusion inhibitors against SARS-CoV-2, and a C-terminally cholesterol-conjugated HR2 peptide, termed IPB02, was identified with the most potent activity.^4^ To gain a more efficient candidate for clinical development, here we dedicated to optimize IPB02 by structural engineering. Five new lipopeptides were designed with IPB02 as a template (Fig. 1b) and their structural properties were first characterized by circular dichroism (CD) spectroscopy. IPB02V1 was created by introducing a flexible PEG8 linker into IPB02 between the peptide sequence and cholesterol molecule; unexpectedly, the introduction fully abolished the α-helicity of the inhibitor (Fig. 1c). With an intention to improve the solubility and binding affinity, a hydrophilic amino acid, aspartic acid (D) or glutamic acid (E), was added to the N-terminus of IPB02V1 respectively; interestingly, the resultant IPB02V2 and IPB02V3 exhibited largely recovered α-helicity. Substitution of two leucine (L) residues for isoleucine (I) in IPB02V3 further generated IPB02V4 with higher helical contents; however, IPB02V5, which adopted four N-terminal amino acids corresponding to the HR2 of MERS-CoV, displayed low α-helicity. When thermal stability of the lipopeptides was measured, IPB02, IPB02V3, and IPB02V4 had melting temperature (*T*_*m*_) of 65, 58, and 67 °C, respectively, whereas the *T*_*m*_ of IPB02V1, IPB02V2, and IPB02V5 could not be determined owing to their low α-helicity (Fig. 1d). We next characterized the IPB02 derivatives for their interactions with a target mimic peptide (HR1P) from the HR1 of SARS-CoV-2. All lipopeptides interacted with HR1P to form α-helical complexes: while the IPB02V2 and IPB02V3 complexes displayed *T*_*m*_ of 51 and 58 °C, respectively, the IPB02, IPB02V1, IPB02V4, and IPB02V5 complexes had a *T*_*m*_ of 46 or 47 °C respectively, indicating their distinct binding stabilities (Fig. 1e, f). Although IPB02V4 showed the highest α-helicity in both the complex or isolation statuses, IPB02V3 was identified with the most stable interaction with the HR1 peptide.

**Fig. 1:**
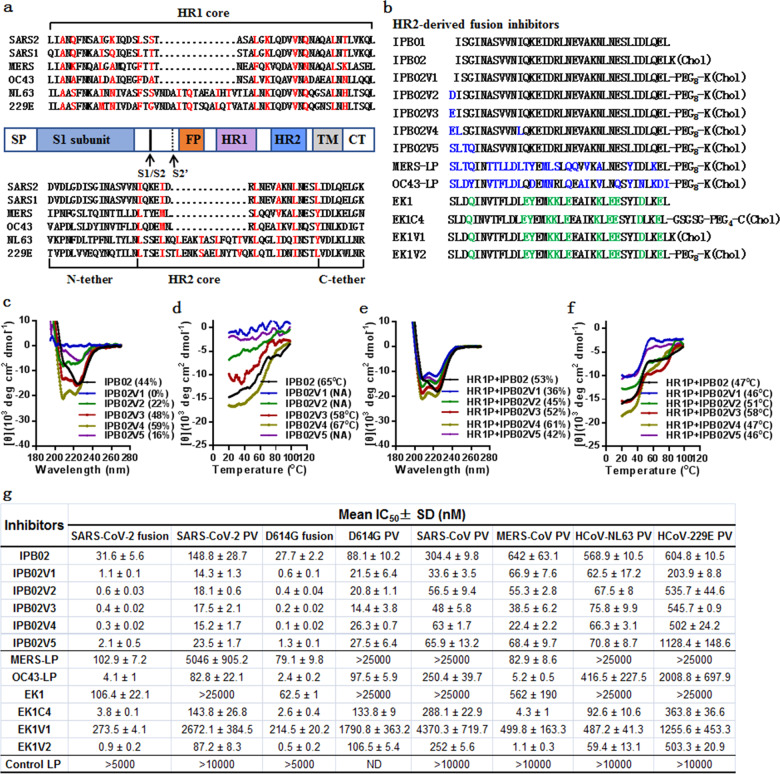
Design and characterization of potent and broad-spectrum fusion inhibitors against divergent human coronaviruses. **a** Schematic diagram of functional domains of the S protein. SP signal peptide, FP fusion peptide, HR1 heptad repeat 1, HR2 heptad repeat 2, TM transmembrane domain, CT cytoplasmic tail. The S1/S2 and S2’ cleavage sites are marked with arrows. The HR1 and HR2 core sequences of representative HCoVs are listed, and the potential residues mediating the HR1–HR2 interactions within the 6-HB core structure are colored in red. **b** HCoV HR2-derived fusion inhibitor peptides and lipopeptides. The residues marked in blue indicate the amino acid changes relative to the template sequence in IPB02. The residues in green indicate the amino acid changes relative to the template sequence in OC43-LP. Chol cholesterol, PEG_8_ 8-unit polyethylene glycol, PEG_4_ 4-unit polyethylene glycol, GSGSG a flexible amino acid linker. **c**–**f** Secondary structure and binding stability of IPB02-based fusion inhibitor lipopeptides. The α-helicity and thermostability of lipopeptides alone (**c**, **d**) or in complexes with the SARS-CoV-2 HR1 target mimic peptide HR1P (**e**, **f**) were determined by CD spectroscopy, with the final concentration of each lipopeptide being 10 μM. The experiments were performed two times, and representative data are shown. **g** The inhibitory activity of various fusion inhibitors against SARS-CoV-2 and other human CoVs. Both dual-split-protein (DSP)-based cell–cell fusion assay and pseudovirus (PV)-based single infection assay were repeated three times, and data are expressed as the means ± standard deviations (SD). D614G represents the SARS-CoV-2 S protein-bearing D614G mutation

We were intrigued to know whether the peptide engineering optimized the inhibitors. A dual-split-protein (DSP)-based cell fusion assay was first applied to determine the inhibitory activity of IPB02 derivatives. As shown in Fig. 1g and Supplementary Fig. S1, IPB02 inhibited the SARS-CoV-2 S-mediated cell–cell fusion with a 50% inhibitory concentration (IC_50_) of 31.6 nM, whereas the IPB02 derivatives showed IC_50_ values ranging from 0.3 to 2.1 nM, indicating 15–105-fold increased potencies. When a single-cycle infection assay was applied, the IPB02 derivatives potently inhibited the SARS-CoV-2 pseudovirus infection in 293T/ACE2 or Huh-7 cells with markedly reduced IC_50_ values either. Recent studies demonstrated that SARS-CoV-2 bearing a spike D614G mutation had become a globally dominant circulating form. Herein, we verified that the D614G mutation did render the S protein with enhanced activities to mediate cell fusion and virus entry, but it did not significantly impact the activity of the inhibitors (Fig. 1g and Supplementary Fig. S2). We further generated a panel of pseudoviruses with the S proteins of SARS-CoV, MERS-CoV, HCoV-229E, and HCoV-NL63 and used in the single-cycle infection assays. Surprisingly, it was found that the lipopeptides exhibited high potency in inhibiting all the tested pseudoviruses (Fig. 1g and Supplementary Fig. S3). None of the IPB02 derivatives at a concentration of 10 μM displayed obvious cytotoxicity in both 293T/ACE2 and Huh-7 cells (Supplementary Fig. S4), indicating their high therapeutic indices.

We wondered whether MERS-CoV-specific fusion inhibitors also have broad-spectrum anti-CoV activity. A lipopeptide termed MERS-LP was therefore synthesized and characterized. As shown in Fig. 1g and Supplementary Fig. S5, MERS-LP inhibited the MERS-CoV pseudovirus infection in Huh-7 cells with an IC_50_ of 89.2 nM, which was even less effective than the IPB02 derivatives. Moreover, MERS-LP only displayed very weak activity in inhibiting SARS-CoV-2 and no cross-inhibition was observed on the SARS-CoV, HCoV-NL63, and HCoV-229E pseudoviruses. Recently, the HCoV-OC43 HR2-derived peptide EK1 and its lipid-derivative EK1C4 were reported as pan-CoV fusion inhibitors.^5^ To reveal the structure–activity relationship of various CoV fusion inhibitors, we further synthesized the lipopeptide OC43-LP with the native HR2 sequence of HCoV-OC43. For comparison, EK1 and EK1C4, as well as two new EK1-derivatives (EK1V1 and EK1V2) per our design strategy, were together produced and characterized. Interestingly, OC43-LP exhibited high inhibitory potencies against all CoVs being tested; especially, it was a highly active inhibitor of MERS-CoV (Fig. 1g and Supplementary Fig. S5). Herein, the unconjugated EK1 peptide only showed weak inhibition on the SARS-CoVs-mediated fusion and MERS-CoV pseudovirus, and no activity was observed against other pseudoviruses even at a high concentration (25,000 nM); nonetheless, the EK1C4 lipopeptide showed comparable potencies with OC43-LP in inhibiting SARS-CoV-2, SARS-CoV, and MERS-CoV. While EK1V1 had poor activity against divergent human CoVs, EK1V2, which was designed analogous to the IPB02 derivatives, displayed dramatically increased anti-CoV potency. In highlight, EK1V2 showed a comparable potency with the PB02 derivatives in blocking the SARS-CoV-2 cell fusion and was the most potent inhibitor of MERS-CoV pseudovirus. The structural properties of four representative MERS-LP and OC43-LP HR2-based inhibitors were also characterized by CD spectroscopy. MERS-LP and OC43-LP alone displayed a small number of helixes, whereas EK1C4 and EK1V2 had significantly increased helixes with *T*_*m*_ of 57 and 61 °C, respectively (Supplementary Fig. S6a, b). All of them interacted with HR1P to form helical complexes, with MERS-LP, OC43-LP, and EK1C4 having enhanced α-helicity (Supplementary Fig. S6c, d). In contrast to the lipopeptides alone, the MERS-LP and OC43-LP complexes had *T*_*m*_ of 47 and 63 °C, respectively, while the EK1C4 and EK1V2 complexes had *T*_*m*_ with minor changes. The interactions of various inhibitors with HR1P were also analyzed by N-PAGE. As shown in Supplementary Fig. S6e, the positively charged peptide HR1P migrated up and off the gel, while an inhibitor alone and its complex displayed specific bands because of their net negative charges.

In conclusion, our studies have offered ideal candidates for the development of potent, broad-spectrum anti-CoV therapeutics and valuable tools for exploiting the mechanism of viral membrane fusion.

## Supplementary information

Supplementary Materials-R1-new

## Data Availability

All data are fully available without restriction.
