# Supplementary material for: SARS-CoV-2-derived fusion inhibitor lipopeptides exhibit highly potent and broad-spectrum activity against divergent human coronaviruses
Source: Signal Transduct Target Ther. 2021 Aug 3;6:294. doi: 10.1038/s41392-021-00698-x (PMC8330190; doi:10.1038/s41392-021-00698-x)
Supplement: Supplementary file 1 — Supplementary Materials-R1-new [file 41392_2021_698_MOESM1_ESM.docx]

Supplementary Materials for

**SARS-CoV-2 derived fusion inhibitor lipopeptides exhibit highly potent and broad-spectrum activity against divergent human coronaviruses**

Yuanmei Zhu, Danwei Yu, Yue Hu, Tong Wu, Huihui Chong, Yuxian He

Correspondence to: yhe@ipb.pumc.edu.cn

**This file includes:**

Fig. S1 Inhibitory activity of IPB02-based lipopeptides against SARS-CoV-2

Fig. S2 Inhibitory activity of IPB02-based lipopeptides against the SARS-CoV-2 D614G mutant

Fig. S3 Broad-spectrum inhibitory activity of IPB02-based lipopeptides against divergent human CoVs

Fig. S4 Cytotoxicity of IPB02-based fusion inhibitor lipopeptides

Fig. S5 Inhibitory activity of the MERS-CoV and HCoV-OC43 HR2-derived fusion inhibitors against divergent human CoVs

Fig. S6 Biophysical characterization of various fusion inhibitory lipopeptides

Material and Methods

Supplemental References


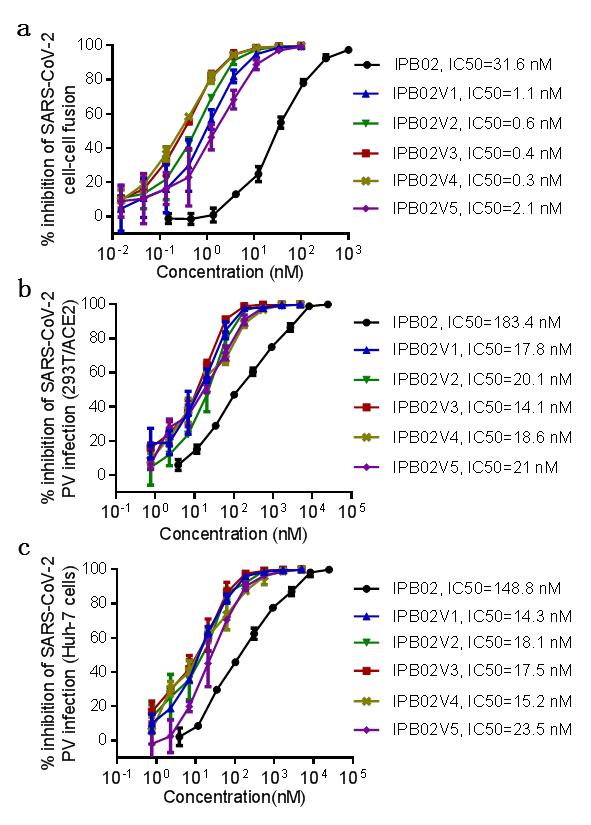


**Fig. S1** **Inhibitory activity of IPB02-based lipopeptides against SARS-CoV-2. a** The inhibitory activity of IPB02 derivatives against the SARS-CoV-2 S protein-mediated cell-cell fusion determined by a DSP-based cell fusion assay. **b**-**c** The inhibitory activity of IPB02 derivatives against the SARS-CoV-2 pseudovirus infection in 293T/ACE2 cells (**b**) or Huh-7 cells (**c**) determined by a single-cycle infection assay. The experiments were repeated three times, and data are expressed as the means ± standard deviations (SD).


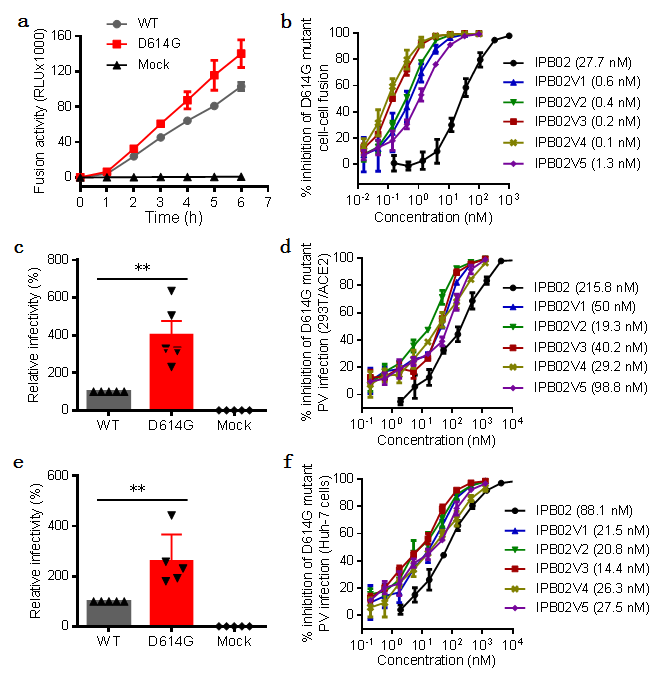


**Fig. S2** **Inhibitory activity of IPB02-based lipopeptides against the SARS-CoV-2 D614G mutant. a** S protein-mediated ell-cell fusion activity; **b** Inhibition of the D614G S mutant-mediated cell fusion by IPB02 derivatives; **c-d** Infectivity of the D614G mutant pseudovirus in 293T/ACE2 cells and its inhibition by IPB02 derivatives; **e-f** Infectivity of the D614G mutant pseudovirus in Huh-7 cells and its inhibition by IPB02 derivatives. Both DSP-based cell fusion assay and PV-based single-cycle infection assay were performed three times, and data are expressed as means ± SD while the mean IC_50_ values are shown in parentheses. For comparing the infectivity of the wild-type (WT) SARS-CoV-2 and D614G mutant pseudoviruses, the virions were normalized to a fixed amount by p24 antigen, the luciferase activity (RLU) of WT was treated as 100% and the relative infectivity of D614G mutant was calculated accordingly. Statistical comparison was conducted by *t*-test (**, *P* < 0.01).


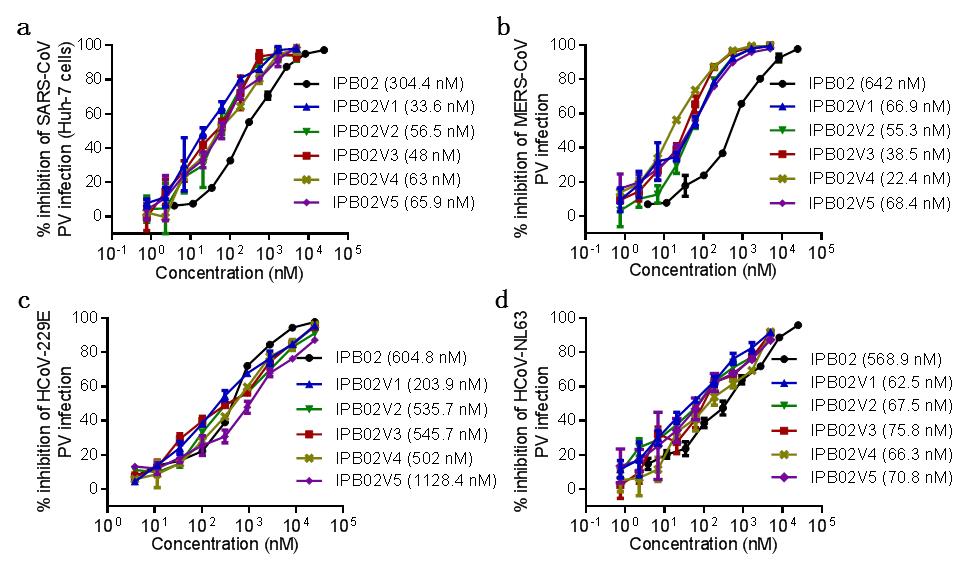


**Fig. S3 Broad-spectrum inhibitory activity of IPB02-based lipopeptides against divergent human CoVs.** The inhibitory activity of IPB02 derivatives against the SARS-CoV (**a**), MERS-CoV (**b**), HCoV-NL63 (**c**) and HCoV-229E (**d**) pseudovirus infections in Huh-7 cells was determined by single-cycle infection assays. The experiments were repeated three times, and data are expressed as means ± SD while the mean IC_50_ values are shown in parentheses.


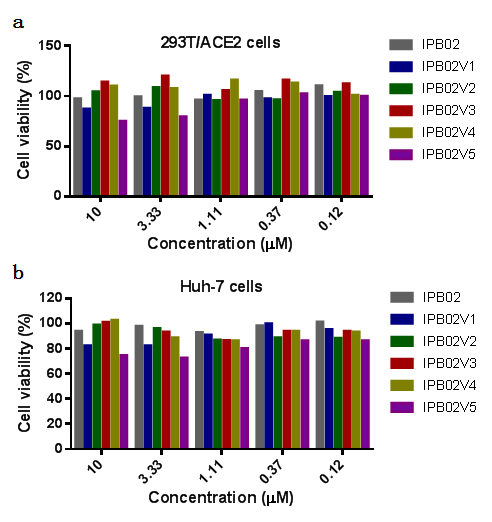


**Fig. S4 Cytotoxicity of IPB02-based fusion inhibitor lipopeptides.** The cytotoxicity of inhibitors on 293T/ACE2 (**a**) and Huh-7 cells (**b**) was measured by a CellTiter 96 AQueous One Solution cell proliferation assay. The experiments were repeated two times and obtained consistent results, and represent data are shown.

**
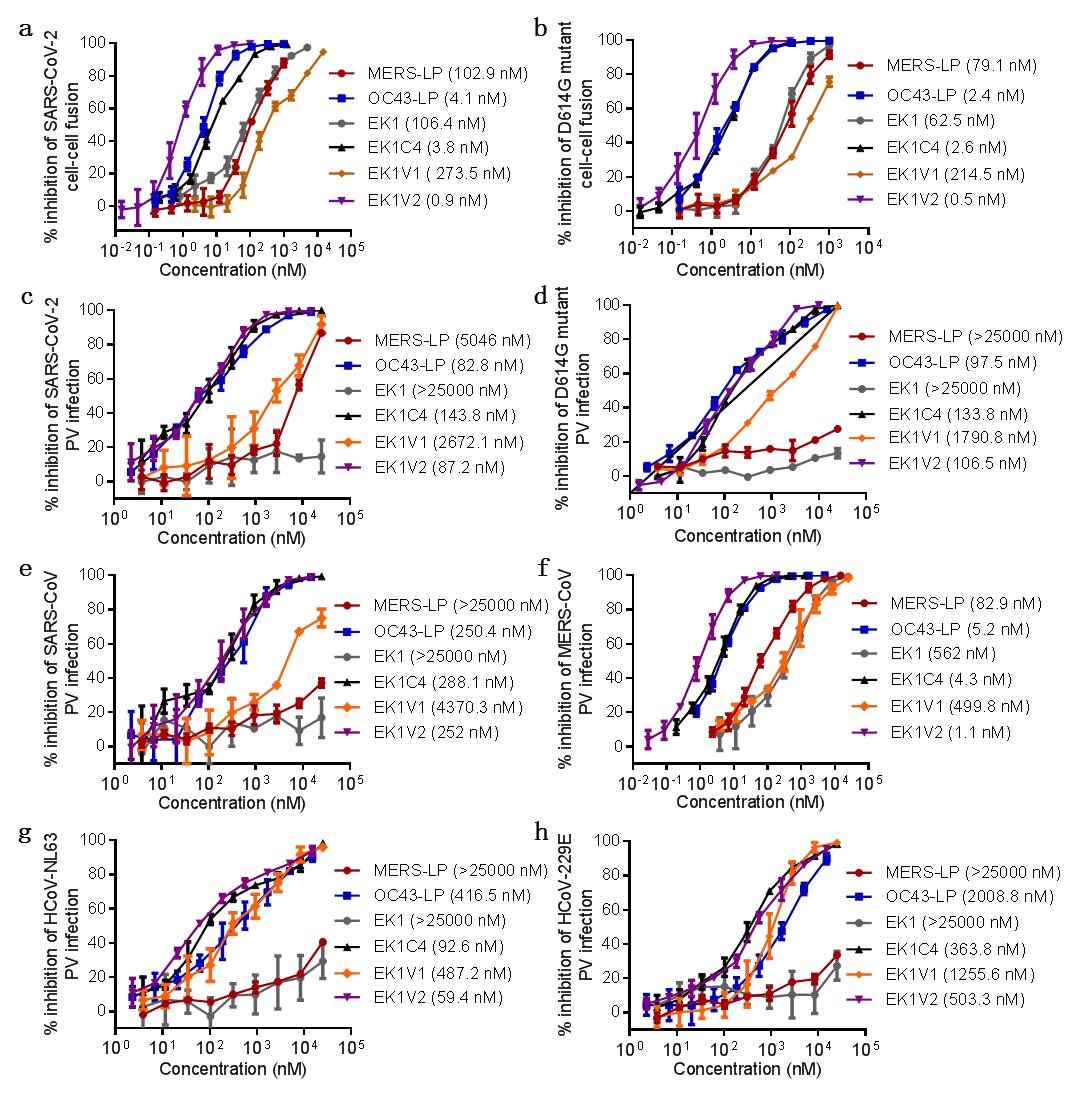
Fig. S5. Inhibitory activity of the MERS-CoV and HCoV-OC43 HR2-derived fusion inhibitors against divergent human CoVs. a-b** The inhibitory activity of various fusion inhibitors against the SARS-CoV-2 (**a**) and D614G mutant (**b**) S protein-mediated cell-cell fusion was determined by DSP-based cell fusion assays. **c-h** The inhibitory activity of the inhibitors against the pseudovirus infections of SARS-CoV-2 (**c**), D614G mutant (**d**), SARS-CoV (**e**), MERS-CoV (**f**), HCoV-NL63 (**g**), and HCoV-229E (**h**) in Huh-7 cells was determined by single-cycle infection assays. The experiments were repeated three times, and data are expressed as means ± SD while the mean IC_50_ values are shown in parentheses.

**
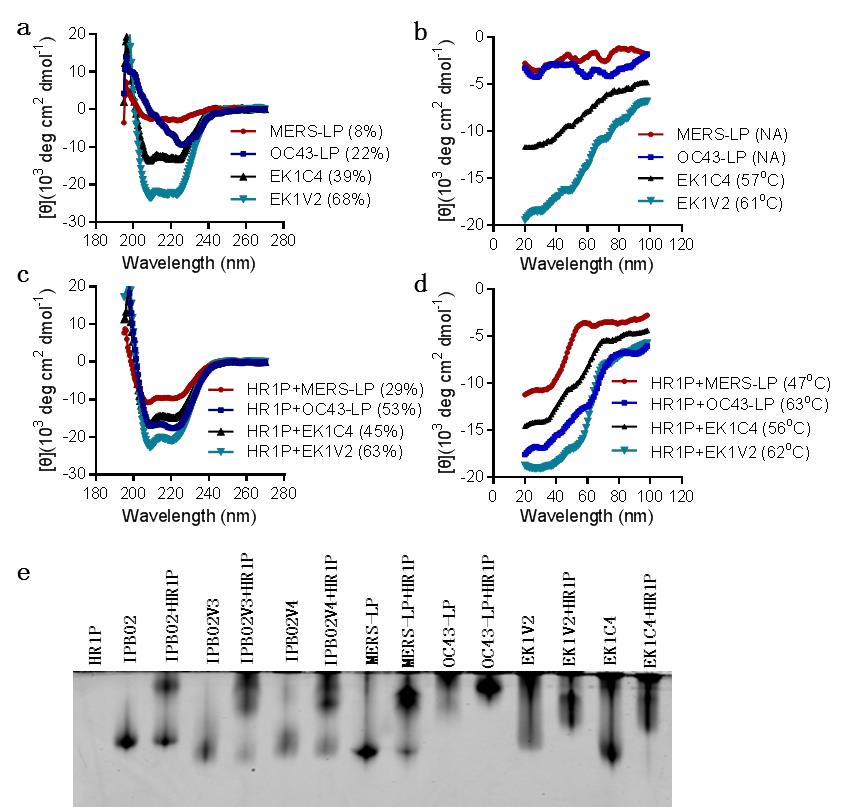
**

**Fig. S6 Biophysical characterization of various fusion inhibitory lipopeptides.** The α-helicity and thermostability of four MERS-CoV and HCoV-OC43 HR2-derivatived lipopeptides alone (**a** and **b**) or in complexes with the SARS-CoV-2 HR1 peptide HR1P (**c** and **d**) were determined by CD spectroscopy, with the final concentration of each lipopeptide being 10 μM. (**e**) Visualization of the interactions between the lipopeptide inhibitors and a target mimic peptide HR1P by N-PAGE analysis. Each peptide was used at a final concentration of 40 μM. Both CD spectroscopy and N-PAGE were repeated two times, and representative data are shown.

**Material and Methods**

**Peptide synthesis and cholesterol conjugation**

Peptides and lipopeptides were produced according to the protocols as described previously.[^1^](#_ENREF_1) Briefly, a peptide was synthesized on rink amide 4-methylbenzhydrylamine (MBHA) resin using a standard solid-phase 9-flurorenylmethoxycarbonyl (FMOC) method. All peptides were acetylated at the N-terminus prior to resin cleavage, followed by purification by reverse-phase high-performance liquid chromatography (HPLC) and characterized with mass spectrometry. The lipidated peptides except EK1C4 were prepared by amidation of a C-terminal lysine side chain with cholesteryl succinate monoester; EK1C4 was prepared by chemoselective thioether conjugation between the peptide containing a C-terminal cysteine residue and bromoacetic acid cholesterol.

**Cell-cell fusion assay**

A dual-split-protein (DSP)-based fusion cell-cell assay was used to measure SARS-CoV-2 S protein-mediated cell-cell fusion activity and the inhibitory activity of peptides, as described previously.[^2^](#_ENREF_2) In brief, a total of 1.5×10^4^ effector cells (HEK293T) were seeded in a 96-well plate and 1.5×10^5^/ml target cells (293T/ACE2) were seeded in a 10-cm culture dish, and then the cells were incubated at 37^o^C. On the next day, the effector cells were cotransfected with a plasmid expressing the SARS-CoV-2 S protein and a DSP_1-7_ plasmid, the target cells were transfected with a DSP_8-11_ plasmid, and then the cells were incubated at 37^o^C. After 24 h, a serially 3-fold diluted peptide was added to the effector cells and the cells were incubated for 1 h; the target cells were resuspended at at 3×10^5^/ml in prewarmed culture medium containing EnduRen live cell substrate (Promega) at a final concentration of 17 ng/ml and incubated for 30 min. Then, 3×10^4^ of target cells were transferred to the effector cells and the mixture of cells were spun down to facilitate cell-cell contact. Luciferase activity was measured at different time points using luciferase assay reagents and a luminescence counter (Promega, Madison, WI, USA), and 50% inhibitory concentration (IC_50_) was calculated as the final cell culture concentration of an inhibitor that caused a 50% reduction in relative luminescence units (RLU) compared to the level of the virus control subtracted from that of the cell control.

**Single-cycle infection assay**

The infectivity and inhibition of pseudoviruses (SARS-CoV-2, SARS-CoV, MERS-CoV, HCoV-NL63, and HCoV-229E) in HEK293T cells that overexpress human ACE2 (293T/ACE2) or Huh-7 cells were determined by a single-cycle infection assay as described previously.[^2^](#_ENREF_2) To generate pseudoviruses, HEK293T cells were cotransfected with a backbone plasmid (pNL4-3.luc.RE) that encodes an Env-defective, luciferase reporter-expressing HIV-1 genome and an S protein-expressing plasmid. After 48 h, cell culture supernatants containing virus particles were harvested, filtrated, and stored at -80^o^C. To detect the inhibitory activity of peptides, pseudoviruses were mixed with an equal volume of a serially 3-fold diluted peptide and incubated at 37 ^o^C for 30 min. The mixture was then added to 293T/ACE2 or Huh-7 cells at a density of 10^4^ cells/100 μl per plate well. After incubation at 37 ^o^C for 48 h, the cells were harvested and lysed in reporter lysis buffer, and luciferase activity was measured and IC_50_ values were calculated as described above.

**Site-directed mutagenesis**

The SARS-CoV-2 spike D614G mutant was generated by site-deirected mutagenesis as described previously.[^3^](#_ENREF_3) Brieﬂy, two primers were designed to contain specific mutation and occupied the same starting and ending positions on the opposite strands of a wild-type (WT) *S*-expressing plasmid. DNA synthesis was conducted by PCR in a 50-μl reaction volume using 100 ng of denatured plasmid template, 50 pM upper and lower primers, and 5 U of the high-fidelity polymerase PrimeStar (TaKaRa, Dalian, China). PCR amplification was done for one cycle of denaturation at 98°C for 5 min, followed by 25 cycles of 98°C for 10 s and 68°C for 9 min, with a final extension at 72°C for 10 min. The amplicons were treated with restriction enzyme DpnI for 3 h at 37°C, and DpnI-resistant molecules were recovered by transforming *Trans 2*-Blue Chemically Competent Cell (TransGen Biotech, Beijing, China) with antibiotic resistance. The required mutation was confirmed by DNA sequencing.

**CD spectroscopy**

Circular dichroism (CD) spectroscopy was used to detect the secondary structure and thermostability of peptides or peptide complexes as described previously.[^2^](#_ENREF_2) Briefly, a peptide was dissolved in phosphate-buffered saline (PBS; pH 7.2) with a final concentration of 10 μM and incubated at 37 ^o^C for 30 min. CD spectra were obtained on Jasco spectropolarimeter (model J-815) a using a 1 nm bandwidth with a 1 nm step resolution from 195 to 270 nm at room temperature. The spectra were corrected by subtracting a solvent blank, and the α-helical content was calculated from the CD signal by dividing the mean residue ellipticity [*θ*] at 222 nm by with a value of -33,000 deg cm^2^ dmol^-1^, corresponding to a 100% helix. Thermal denaturation was done by monitoring the ellipticity change at 222 nm from 20 to 98^o^C at a rate of 2^o^C/min, and the melting temperature (*T_m_*) was defined as the midpoint of the thermal unfolding transition.

**N-PAGE**

Native-polyacrylamide gel electrophoresis (N-PAGE) was conducted to detect the interactions between an HR2-derived lipopeptide inhibitor and a SARS-CoV-2 HR1-derived target mimic peptide (HR1P) as described previously.[^2^](#_ENREF_2) Briefly, an HR1 peptide was mixed with an HR2 peptide at a final concentration of 40 μM and incubated at 37^o^C for 30 min. Tris–glycine native sample buffer was added to the mixture at a ratio of 1 : 1, and then the mixture was loaded at 25 μl/well onto a 20% Tris-glycine gel (10- by 1.0-mm). Gel electrophoresis was conducted with a 100-V constant voltage at 4 ^o^C for 3 h, and then the gel was stained with Coomassie blue and imaged with a Bio-Rad imaging system (Bio-Rad, Hercules, California, USA).

**Cytotoxicity of inhibitors**

The cytotoxicity of lipopeptide fusion inhibitors on 293T/ACE2 and Huh-7 cells was measured using a CellTiter 96 AQueous One Solution cell proliferation assay (Promega). In brief, 50-μl volumes of lipopeptides at graded concentrations were added to cells, which were seeded on a 96-well tissue culture plate (1 × 10^4^ cells per well). After incubation at 37˚C for 2 days, 20 μl of CellTiter 96 AQueous One solution reagent was added into each well and incubated 2 h at 37˚C. The absorbance was measured at 490 nm using a SpectraMax M5 microplate reader (Molecular Devices, San Jose, CA, USA), and cell viability (percentage) was calculated.

**Supplemental References**

1 Zhu, Y. et al*.* Design and Characterization of Cholesterylated Peptide HIV-1/2 Fusion Inhibitors with Extremely Potent and Long-Lasting Antiviral Activity. *J. Virol.* **93**, e02312-18 (2019).

2 Zhu, Y., Yu, D., Yan, H., Chong, H. & He, Y. Design of Potent Membrane Fusion Inhibitors against SARS-CoV-2, an Emerging Coronavirus with High Fusogenic Activity. *J. Virol.* **94**, e00635-20 (2020).

3 Yu, D. et al*.* Therapeutic Efficacy and Resistance Selection of a Lipopeptide Fusion Inhibitor in Simian Immunodeficiency Virus-Infected Rhesus Macaques. *J. Virol.* **94**, e00384-20 (2020).
